# Neurotrophic Keratopathy and Topical Insulin Therapy: A Case Report

**DOI:** 10.7759/cureus.46242

**Published:** 2023-09-30

**Authors:** Maryum Khilji, Shafiq Tanveer, Fahd Zafar Khan, Dilawar Ali Yazdan, Ayesha Khilji

**Affiliations:** 1 Ophthalmology, Khyber Medical College/Khyber Teaching Hospital, Peshawar, PAK; 2 Community Medicine, Khyber Medical College, Peshawar, PAK; 3 Internal Medicine, Hayatabad Medical Complex, Peshawar, PAK

**Keywords:** therapeutics, cornea, topical insulin therapy, herpetic keratitis, corneal ulcer, neurotrophic keratopathy

## Abstract

Neurotrophic keratopathy is a rare disorder caused by the loss of corneal sensation. It is characterized by persistent epithelial defects, corneal ulceration, and, ultimately, corneal perforation if not managed in a timely manner. The management includes aggressive lubrication, prophylactic topical antibiotics, therapeutic contact lenses, tarsorrhaphy, and amniotic membrane transplantation. Some novel therapeutic options are also available, one of which is topical insulin therapy. We report the clinical course of a patient with neurotrophic keratopathy that was successfully treated with topical insulin.

A 64-year-old male presented to our outpatient department with a three-month history of painless blurring of vision following prior episodes of herpetic keratitis. Ocular examination showed a bilateral reduction in corneal sensations, bilateral corneal opacities, and a corneal ulcer in the left eye. He was diagnosed as a case of neurotrophic keratopathy secondary to prior herpetic keratitis. He was then treated with topical and oral acyclovir along with topical insulin drops. There was a remarkable improvement in his condition after a month with a reduction in the size of the ulcer and, after two months, the ulcer was completely re-epithelialized. This case report illustrates the use of topical insulin in the initial management of neurotrophic keratopathy as opposed to its conventional use in refractory neurotrophic corneal ulcers.

## Introduction

Neurotrophic keratopathy is a rare disease that occurs due to the loss of corneal sensation, resulting in persistent epithelial defects, corneal ulcers, and if left untreated, corneal melting and perforation. It affects five in 10,000 people and is classified as an orphan disease [1]. Damage to the trigeminal nerve leads to impaired corneal sensation that in turn causes abnormal blink reflex, tear film abnormalities, epithelial breakdown, and impaired wound healing [2,3]. Mackie classification is generally used to grade the severity of the disease. Stage 1 is characterized by a cloudy cornea, corneal edema, and superficial punctate keratopathy. Stage 2 includes persistent/recurrent epithelial defect (sometimes with a rolled edge), Descemet membrane folds, and stromal swelling. Stage 3 includes frank corneal ulcer that may lead to stromal melting and, ultimately, corneal perforation [4].

There are multiple causes of neurotrophic keratopathy, all of them leading to impairment of trigeminal nerve function [4]. Some of the common causes are post-infectious, i.e., ocular infection with *Herpes simplex* virus (HSV) or *Varicella zoster* virus (VZV), ocular surgery (e.g., LASIK, cataract surgery, keratoplasty), long-term contact lens use, chemical and thermal injuries, surgery for trigeminal neuralgia, extensive pan-retinal photocoagulation, intracranial tumors (acoustic neuroma, meningioma, etc.), diabetic neuropathy, topical anesthetic abuse, and prolonged use of medications such as beta-blockers and non-steroidal anti-inflammatory drugs (NSAIDs) [2,5].

Early diagnosis, treatment, and monitoring are crucial to prevent the debilitating effects of neurotrophic keratopathy. The evaluation includes taking a thorough ophthalmic and systemic history, performing an ocular exam, and checking for trigeminal nerve function. The corneal sensation can be grossly checked with a fine cotton wisp but can be quantitatively measured by the Cochet-Bonnet aesthesiometer [1]. The treatment depends on the stage of the disease but usually involves aggressive lubrication (preferably with preservative-free artificial tears) and prevention of secondary bacterial infection by topical antibiotics [4]. More severe epithelial defects need therapeutic contact lenses, temporary tarsorrhaphy, conjunctival flap surgery, and amniotic membrane transplantation. Corneal neurotization surgery is another option [6].

Some novel therapies include recombinant human nerve growth factor (rhNGF), substance P, autologous serum drops, umbilical cord serum, and topical insulin eye drops [7]. This report describes a case of neurotrophic keratopathy that was treated with topical insulin eye drops with positive results.

## Case presentation

A 64-year-old male presented to our outpatient department at Khyber Teaching Hospital with the complaint of painless blurring of vision in both eyes. It had started a year ago and progressively worsened over the past three months. It was not associated with any pain, watering, or photophobia. The systemic review was unremarkable. The patient was non-diabetic and non-hypertensive. The rest of the past medical history was unremarkable. Past surgical history was significant for bilateral cataract surgery 10 years ago. He was not using any medication at the time of the visit. However, he had used topical acyclovir ointment 5% five times a day for 15 days until approximately two weeks prior to his visit to our hospital. It was reportedly prescribed by an ophthalmologist but the record was unavailable.

Past ocular history revealed an episode of painful decrease in vision in both eyes the past year that had occurred shortly after applying kohl in his eyes. He had been prescribed antibiotic-steroid eye drops (chloramphenicol-dexamethasone) by a local practitioner, resulting in improvement. However, subsequent evaluation by an ophthalmologist revealed bilateral corneal opacities and high intraocular pressure (IOP), likely due to steroid use. Topical beta-blocker eye drops (timolol 0.5% two times a day for two months) were initiated. Optical coherence tomography of the retinal nerve fiber layer (OCT-RNFL) showed no abnormalities. The patient noted improvement, but three months later, he had a recurrence of symptoms in the right eye only. He was diagnosed with right herpetic keratitis and started on oral acyclovir 800 mg five times a day and topical acyclovir ointment 5% five times a day in the right eye for 10 days, leading to the resolution of the condition. Two months later, the patient reported a recurrence of symptoms in the left eye and was diagnosed with left herpetic keratitis. He was treated with oral acyclovir 800 mg five times a day and topical acyclovir ointment 5% five times a day in the left eye for 10 days. Thereafter, the patient was unavailable for subsequent follow-up visits, but he presented to us again after a year, now with painless blurring of vision in both eyes.

**Figure 1:**
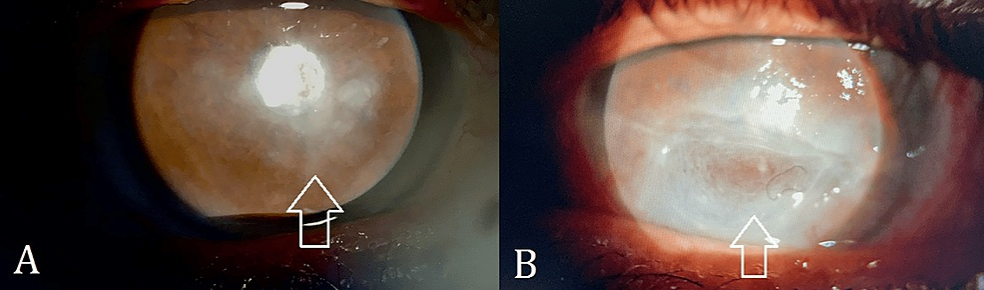
Slit lamp photograph (anterior segment, direct illumination) of the right eye The images show (A) central corneal opacity of the left eye and (B) inferior corneal ulcer with surrounding opacity and thinning

**Figure 2:**
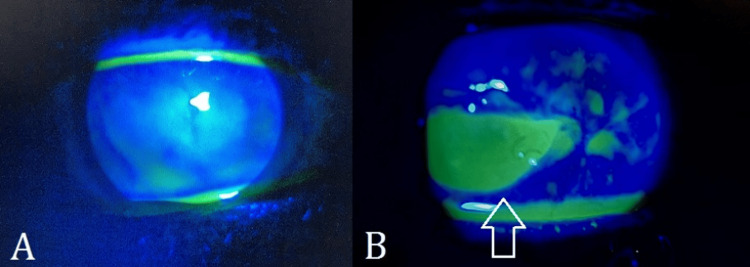
Slit lamp photographs (anterior segment, direct illumination with cobalt blue filter) after the instillation of sodium fluorescein in both eyes The right eye (A) shows no staining while the left eye (B) shows staining of the corneal ulcer

On ocular examination, his visual acuity was 4/60 in the right eye, and hand movements in the left eye with no further improvement. The pupils were round, regular, and reactive to light with no relative afferent pupillary defect (RAPD). The extraocular movements were normal. The adnexa showed no abnormalities. The conjunctiva in the right eye was mildly hyperemic while the left eye showed ciliary flush. Corneal sensation was grossly assessed using a cotton wisp; it was found to be reduced in both eyes. The right cornea showed an opacity measuring 5 x 7 mm over the visual axis (Figure 1A), whereas the left cornea showed a boat-shaped corneal ulcer measuring 4 x 6 mm in the inferior aspect surrounded by an area of corneal thinning and opacification (Figure 1B). There was corneal neovascularization extending to the inferior margin of the ulcer from the inferior limbus in the left eye. No staining was observed in the right eye (Figure 2A), while the ulcer in the left eye exhibited staining after the instillation of sodium fluorescein (Figure 2B). The anterior chambers were deep and quiet. The patient was pseudophakic in both eyes. The IOP was 16 mmHg in the right eye and 10 mmHg in the left eye. Fundus examination could not be performed due to bilateral corneal opacities. B-scan ultrasonography was performed to rule out any posterior segment pathology, and it was normal. All baseline systemic investigations were also normal.

The patient was diagnosed as a case of neurotrophic keratitis secondary to prior herpetic keratitis. Treatment was initiated with oral acyclovir 400 mg twice a day, topical acyclovir ointment 5% five times a day, and topical insulin drops six times a day (prepared by mixing 1 IU/ml of regular insulin in 10 ml of artificial tears with polyvinyl alcohol) for a month.

**Figure 3:**
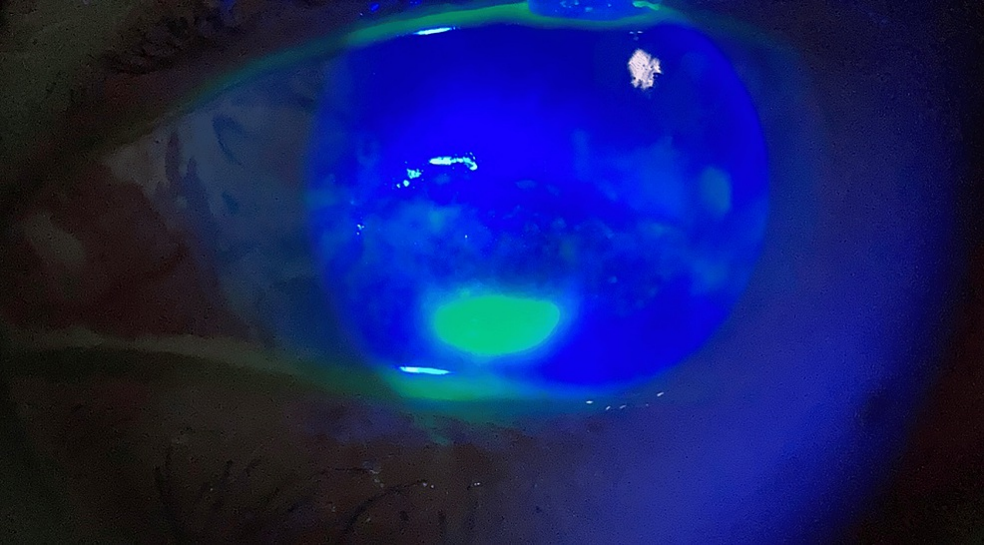
Slit lamp photograph (anterior segment, direct illumination with cobalt blue filter) of the left eye following staining with sodium fluorescein There is a decrease in the size of the corneal ulcer after a month of treatment

**Figure 4:**
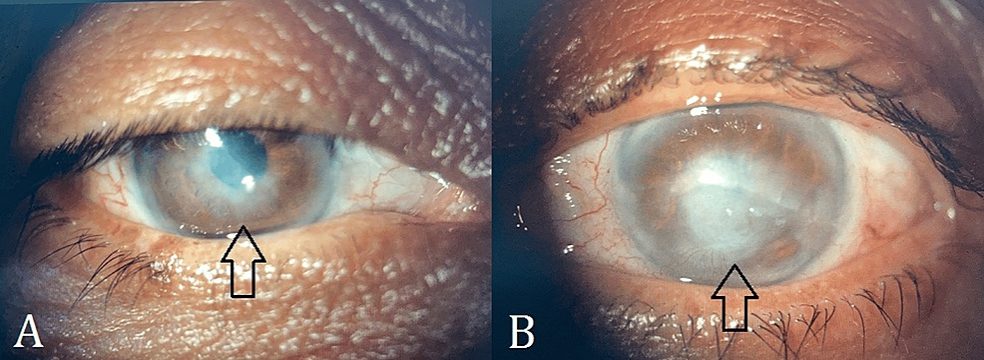
Slit lamp photograph (anterior segment, direct illumination) of the right eye The images show (A) decreased haziness in the left eye and (B) a completely healed corneal ulcer after two months of treatment

**Figure 5:**
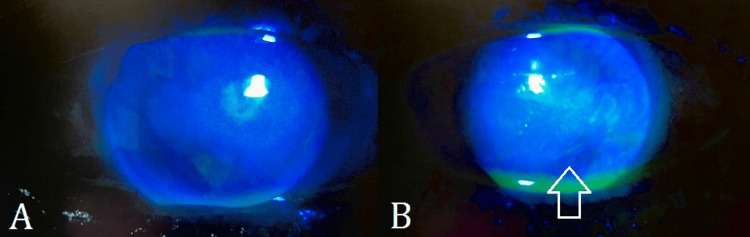
Slit lamp photograph (anterior segment, direct illumination with cobalt filter) of the right eye (A) and the left eye (B) after staining with sodium fluorescein Note the completely healed corneal ulcer (B) after two months of treatment

Upon follow-up after a month, the patient showed marked improvement subjectively and objectively. The visual acuity improved to 6/60 in the right eye and 1/60 in the left eye. The ciliary flush in the left eye had resolved. The opacity in the right eye maintained its original size but it appeared to have cleared slightly. The corneal ulcer in the left eye exhibited a decrease in size (1 x 2 mm) (Figure 3). Oral acyclovir was discontinued; topical acyclovir ointment was continued and topical insulin was tapered to five times per day. The patient was re-evaluated after a month, revealing complete healing of the ulcer (Figures 4A, 4B) with no staining (Figures 5A, 5B). This highlights the promising role of topical insulin in the treatment of neurotrophic keratitis secondary to herpetic keratitis.

## Discussion

In this case report, we highlight the role of low-dose topical insulin in the treatment of neurotrophic keratopathy secondary to prior HSV infection with rapid healing and no significant side effects. The role of insulin in the treatment of neurotrophic keratopathy is not well understood. Insulin is found in the tear film and insulin receptors along with insulin-like growth factor 1 (IGF-1) receptors are found in the cornea and conjunctiva [8]. Diabetics suffer more from dry eye syndrome (DES) as compared to the non-diabetic population, suggesting the role of insulin in maintaining the ocular surface [9].

There have been a few studies on the role of topical insulin in the treatment of corneal pathologies, most of them showing the use of insulin in neurotrophic ulcers refractive to other medical and surgical therapies. However, to our knowledge, our study is the only one to demonstrate the use of topical insulin as an early treatment option for neurotrophic keratopathy. Wang et al. [10] described the use of topical insulin in six cases of neurotrophic keratopathy, resulting in full re-epithelialization in all six cases within a short period with no debilitating side effects. Tong et al. [11] also demonstrated the use of topical insulin for the treatment of bilateral neurotrophic ulcers in a patient with poorly controlled diabetes, but it involved a higher concentration than ours (25 IU/ml). One limitation of our study is the concomitant use of acyclovir along with insulin for treatment.

## Conclusions

Topical insulin (with a low dose of 1 IU/ml) shows promising results as an early, low-cost, and effective treatment for neurotrophic keratopathy secondary to HSV infection. The use of topical insulin therapy for this condition is still considered experimental and not yet a standard or widely accepted treatment; hence, further studies are needed to establish the role of insulin monotherapy as a first-line treatment for neurotrophic keratopathy. Future research avenues include conducting clinical trials to investigate the long-term effectiveness and safety of topical insulin therapy, assessment of long-term visual outcomes, and conducting studies to compare topical insulin therapy with other pre-existing treatments.
